# Surface excitations in electron spectroscopy. Part I: dielectric formalism and Monte Carlo algorithm

**DOI:** 10.1002/sia.5175

**Published:** 2012-12-26

**Authors:** F Salvat-Pujol, W S M Werner

**Affiliations:** Institut für Angewandte Physik, Vienna University of TechnologyWiedner Hauptstraße 8-10, A 1040, Vienna, Austria

**Keywords:** surface excitations, dielectric formalism, Monte Carlo simulation of electron transport, differential inelastic inverse mean free path (DIIMFP), stopping power, reflection electron energy loss spectroscopy (EELS, REELS), X-ray photoelectron spectroscopy (XPS), Auger-electron spectroscopy (AES)

## Abstract

The theory describing energy losses of charged non-relativistic projectiles crossing a planar interface is derived on the basis of the Maxwell equations, outlining the physical assumptions of the model in great detail. The employed approach is very general in that various common models for surface excitations (such as the specular reflection model) can be obtained by an appropriate choice of parameter values. The dynamics of charged projectiles near surfaces is examined by calculations of the induced surface charge and the depth- and direction-dependent differential inelastic inverse mean free path (DIIMFP) and stopping power. The effect of several simplifications frequently encountered in the literature is investigated: differences of up to 100% are found in heights, widths, and positions of peaks in the DIIMFP. The presented model is implemented in a Monte Carlo algorithm for the simulation of the electron transport relevant for surface electron spectroscopy. Simulated reflection electron energy loss spectra are in good agreement with experiment on an absolute scale. Copyright © 2012 John Wiley & Sons, Ltd.

## Introduction

A quantitative understanding of any spectroscopy technique based on the analysis of reflected, transmitted, or emitted charged projectiles from solid surfaces relies on an accurate description of the inelastic interaction of the projectiles with the target. In the bulk of the solid, the stopping of the projectile can be accurately described within the semiclassical dielectric formalism for infinite media: the presence of the charged projectile perturbs the equilibrium charge distribution of the solid, which becomes polarized and induces an electromagnetic (e.m.) field that acts as a stopping force on the projectile. Within the semiclassical approximation, the Fourier spectrum of the field set up by the projectile is interpreted as a distribution of energy losses and momentum transfers at individual, discrete inelastic interactions. Thus, the semiclassical approximation serves as the link between the classical dielectric description and the quantal nature of inelastic interactions. The presence of an interface, e.g. solid-vacuum or between two solids, imposes strict boundary conditions on the total (and hence also on the induced) e.m. field. Thus, the stopping of the projectile close to the interface is substantially different than in the bulk of the solid: an additional dependency is found on the distance to the interface (at either side) and on the surface crossing direction.

A collective response of the electrons in the surface region of the solid due to the passage of charged projectiles through the interface was predicted in the 1950s by Ritchie^1^ and confirmed experimentally by Powell and Swan.^2^,^3^ A number of models have been developed in the last decades to understand the collective (and single-particle) response.^4^–^23^ Different approaches and approximations are adopted: some models use a classical electrodynamics framework whereas others use many-body quantum theory, some assume a simplified dielectric response of the solid, some are valid for particular trajectories of the projectile (e.g. perpendicular or parallel to the surface). Simplifying mathematical assumptions are often made in order to highlight the relevant physics, to obtain more treatable expressions, and to keep the computation time within reasonable limits. Unfortunately, the effect of the different approximations on the resulting physical description of the stopping of the projectile has not been scrutinized.

It is the purpose of the present work to present a self-contained description of the dynamics of charged projectiles near a planar interface within the semiclassical dielectric formalism, taking special care to avoid further approximations such as those addressed above. A common framework is provided which allows one to compare a subset of models in the literature and to expose the effects of their implied approximations. The calculation procedure presented here has been implemented in a Monte Carlo algorithm for the simulation of reflection-electron-energy-loss spectra. Finally, the quality of the model will be assessed by means of comparisons of simulated reflection electron energy loss spectra (REELS) with experimental REELS on an absolute scale.

This work is structured as follows: In section “Theory”, the dielectric formalism for the stopping of charged projectiles in an infinite medium is briefly reviewed and extended to take into account the presence of a planar interface to another medium. An expression for the differential inelastic inverse mean free path (DIIMFP) of a charged projectile in the vicinity of a planar interface is derived. In section “Results”, the dependence of the derived DIIMFP on the velocity of the projectile, on the distance at each side of the interface, and on the surface crossing direction is examined. Calculated DIIMFPs are compared with those given by another model from the literature, exposing the effects of its implied approximations. Next, a Monte Carlo algorithm for the simulation of electron reflection experiments is described. Simulated spectra are compared in absolute units with experimental spectra. Finally, in section “Conclusions”, the presented results are summarized, and conclusions are drawn.

## Theory

**Figure 1:**
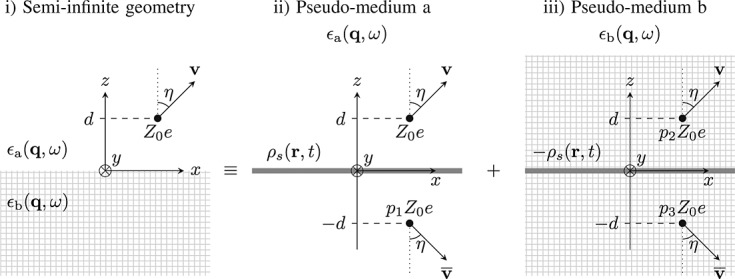
(i) Considered geometry: a non-relativistic projectile of charge *Z*_0_*e* and velocity **v** is at a distance *d* at either side of the interface between two media *ε*_*a*_(**q**,*ω*) and *ε*_*b*_(**q**,*ω*) moving with a polar angle *η*. (ii) and (iii): Schematic representation of the method of image charges for dielectrics. By an appropriate choice of the contributions *p*_1_, *p*_2_, and *p*_3_ (unity or zero) different models found in the literature can be reproduced, such as the surface-reflection model, for which *p*_1_ = *p*_2_ = *p*_3_ = 1 [see text and Eqn (8)].

We consider the geometry depicted in Fig. 1i. A projectile of charge *Z*_0_*e* moves near the interface between two semi-infinite media with dielectric functions *ε*_*a*_(**q**,*ω*) and *ε*_*b*_(**q**,*ω*), where the subscripts *a* and *b* imply above and below the interface, respectively.† For the formulae to apply for electrons, *Z*_0_ must be set to –1. We use a system of Cartesian coordinates where the surface is the plane *z* = 0 and the unit vector along the *z* axis, 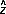, points towards medium *a*. The charged projectile moves in a uniform motion with velocity **v** making an angle *η* with the positive *z* axis. We denote the *z* coordinate of the projectile's position as *d*. There is mirror symmetry with respect to the plane spanned by **v** and the *z* axis, and, furthermore, in the case of normal escape and normal incidence (**v** parallel and opposed to 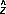, respectively), all physical quantities we derive must be invariant under an arbitrary rotation around the *z* axis. We will use the Gaussian system of units [1/(4*πε*_0_) = 1, where *ε*_0_ is the vacuum permittivity, and *μ*_0_/(4*π*) = 1, where *μ*_0_ is the vacuum permeability] throughout the calculation, unless stated otherwise.

First, the method of image charges will be used to solve Maxwell's equations in order to obtain the total e.m. field set up by the projectile with the appropriate boundary conditions. Next, the e.m. field induced at the position of the projectile will be determined. Finally, an expression for the average energy loss per unit path length (the so-called electronic stopping power) will be derived as a function of the distance to the interface, *d*, the surface crossing angle, *η*, and the speed of the projectile, *v*. The connection between this classical description and the quantized nature of the inelastic interaction of the charged projectile in the solid will be made at the final stage of the calculation by means of the semiclassical approximation: *ħ***q** and *ħω* will be considered as the momentum and energy transfers from the projectile to the solid in an individual inelastic interaction. Here, **q** and *ω* are the Fourier conjugate variables of the position and time variables **r** and *t*, respectively. By strict analogy with the quantal calculation, one may derive not only the average energy loss per unit path length, but also the DIIMFP, a quantity proportional to the probability of undergoing a discrete energy loss between *W* and *W* + d*W* per unit path length.

We will work in the Coulomb gauge, where the vector potential **A**(**r**,*t*) is transverse: ∇ ⋅ **A**(**r**,*t*) = 0. We recall that the electric field in terms of the scalar potential *φ*(**r**,*t*) and the vector potential **A**(**r**,*t*) is given by^25^,^26^


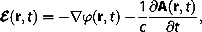
(1)

where *c* is the speed of light in vacuum. As we are concerned with energy losses of relatively slow projectiles (with speeds much less than *c*), the transverse components of the e.m. field can be ignored. Consequently, we take **A** = 0, so that (**B** is thus automatically set to zero):


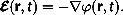
(2)

The effect of this approximation is twofold: (i) Maxwell's equations are formally reduced to the electrostatic case even though the position of the charged projectile and, hence, the induced field vary with time and (ii) the theory does not describe the effect of ‘transverse interactions’, which contribute to the energy loss of relativistic projectiles and cause effects such as transition radiation and Cerenkov radiation. In the case of electrons, this implies that the present calculation is valid for kinetic energies up to a few tens of keV. Retardation effects due to the finite propagation speed of the e.m. field are not accounted for explicitly.

We assume that the perturbation caused by the passage of the projectile through the media is small enough so that the media respond in a linear fashion. For homogeneous media, this is equivalent to assuming that the electric displacement field 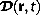 and the electric field are related via the linear relationship


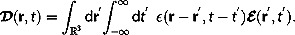
(3)

In Fourier space, this expression becomes


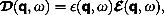
(4)

where *ε*(**q**,*ω*) is the dielectric function of the medium. The linear-response approximation is formally equivalent to a first-order Born approximation in perturbation theory, which is valid for projectile energies of about 20 times the characteristic energies of the active target electrons (ionization energies in the case of inner-shell excitations).^27^ Other estimations in the case of inelastic scattering in solids^15^ suggest a lower bound of valid kinetic energies in the order of 7*E*_*F*_, where *E*_*F*_ is the Fermi energy of the solid. Thus, in the case of electrons, the present calculation is strictly valid for kinetic energies between ∼ 100 eV and ∼ 10 keV.

We recall that the dielectric function gives the response of the solid to the (**q**,*ω*)-Fourier component of an external e.m. field. If the external perturbation is a plane wave with frequency *ω* propagating along a given direction, e.g. the electric field carried by incoming photons in the case of x-ray or UV spectroscopy, the modulus of the electric field is independent of the spatial coordinates and, hence, the response of the solid is characterized by a dielectric function that depends only on the frequency of the wave, *ε*(*ω*). If the perturbation is due to a moving charged projectile, the electrons of the solid around the projectile interact with it via the Coulomb potential, which evidently depends on the spatial coordinates. Thus, the polarization of the material is non-local and is therefore in general a non-trivial function of both **r** and *t*, or **q** and *ω* in Fourier space. Therefore, strictly speaking, in order to describe the dielectric response of a solid to the passage of a charged projectile, a dielectric function of the form *ε*(**q**,*ω*) is needed. This kind of dielectric function is known in the literature as spatially dispersive, owing to the dependency on **q**, namely on **r** in real space.

Finally, we recall that the boundary conditions of the electric field and of the electric displacement field at the interface read^25^


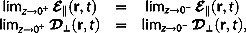
(5)

where the subscripts ‖ and ⊥ indicate, respectively, the components parallel and perpendicular to the surface. These boundary conditions must hold at all times at any point on the surface. They also apply for time-varying fields.^25^

### Method of image charges

Maxwell's equations for electrostatics with a given set of boundary conditions can be formally solved by means of the Green's function formalism.^26^,^25^ The potential is given as an integral relationship within a closed volume involving a kernel of the form 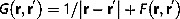, where **r** is the observation point and 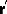 is the source point. The first term of 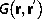 is the potential at **r** due to a point charge at 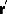. The second term, 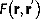, is seen to satisfy the Laplace equation^25^ (∇^2^φ(**r**,*t*)=0, potential in a region of space with no charges) and can therefore be understood as the potential of a system of charges external to the integration volume, chosen in such a way that, together with a source at 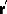, the required boundary conditions are satisfied. The method of image charges for dielectrics,^25^ also known in the literature as the method of extended pseudomedia,^28^,^29^ is a practical approach which is formally equivalent to determining 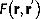, with the benefit of a conceptually simpler development. It consists in finding an alternative representation of the problem in terms of fictitious charge distributions chosen in such a way that the electric field (i) can be trivially found and (ii) verifies the same differential equation (Maxwell's equations) and the same boundary conditions as in the original problem. Such an alternative representation is therefore equivalent to the original problem for all purposes.

We consider two infinite pseudomedia *a* and *b* with bulk dielectric functions *ε*_*a*_(**q**,*ω*) and *ε*_*b*_(**q**,*ε*), respectively (see Fig. 1ii and iii). In order to emulate the effect of the interface in the real problem, fictitious charge distributions will be added to each pseudomedium. Next, the electric field in each pseudomedium, 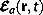 and 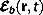, will be determined. The electric field in the semi-infinite geometry will then be given by


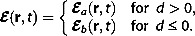
(6)

At this point, the electric field will still contain the fictitious charge distributions explicitly. Next, the boundary conditions (5) will be applied and the fictitious charges shall be completely determined in terms of other parameters and disappear from the equations, thus serving as a convenient means of solving Maxwell's equations satisfying the corresponding boundary conditions at the planar interface. Each infinite pseudomedium exhibits translational invariance: its dielectric response can be described in terms of the bulk dielectric function. Thus, the method of image charges allows us to describe surface effects using bulk properties of the media (!). We point out for completeness that an alternative approach consists in determining the surface dielectric response explicitly.^30^,^31^

The fictitious charge distributions at each pseudomedium will now be specified. The question arises as to whether the electric field is uniquely defined, that is, whether different choices of fictitious charges yield the same electric field. We recall that the electrostatic problem is uniquely determined only for Dirichlet or Neumann boundary conditions^25^ (specifying the potential or the normal derivative of the potential, respectively, everywhere on a closed surface). The boundary conditions, Eqn (5), are neither Dirichlet nor Neumann boundary conditions: only continuity of the parallel component of the electric field and of the normal component of the displacement through the surface is required, not a specific value. We therefore assume that the electric field which satisfies the required conditions is not unique. Other authors make similar statements: Chan *et al*.^11^ state that ‘different choices of fictitious charge distributions correspond to different boundary conditions’ and, similarly, Flores *et al*.^29^ mention that ‘there exists a precise relationship between boundary conditions, physical models of surface scattering, and corresponding surface pseudostimuli.’ Thus, it must be observed that different choices of fictitious charges yield different descriptions of the inelastic scattering of charged projectiles near a surface. See the note after Eqn (8) for a further clarification of this point.

Recall that the velocity of the projectile is given by **v** = (*v*_*x*_,*v*_*y*_,*v*_*z*_). The planar interface *z* = 0 suggests a candidate fictitious charge at each pseudomedium: the specular image charge of the projectile, which moves with a velocity 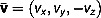. Furthermore, as we will see below, the use of spatially dispersive dielectric functions implies that a fictitious surface charge *ρ*_*s*_(**r**_‖_,*t*) must be added at the plane *z* = 0.^32^,^33^ Thus, we express the charge distributions in pseudomedia *a* and *b* as


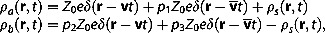
(7)

respectively. Notice the opposite sign of the surface charge in pseudomedium *b* in Eqn (7). This sign is necessary; otherwise, the normal component of the electric displacement field cannot be continuous through the surface.^33^ The scalar parameters *p*_1_, *p*_2_, and *p*_3_ have been introduced to conveniently reproduce different models found in the literature:


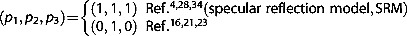
(8)

If the calculation is carried out including the transverse part of the electric field, one finds that the boundary conditions for the transverse part of 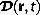 can only be satisfied if the image charge is selected, e.g. (*p*_1_, *p*_2_, *p*_3_) = (1,1,1). It is then seen that this point image charge accounts for a bulk polarization current moving away from the interface as the projectile moves towards it in the medium side. It is implicitly understood that the electrons of the solid bounce back towards the inside of the medium at the interface as they are repelled by the projectile (assuming an electron). This picture corresponds to the specular reflection model.^4^ In the present calculation, where we neglect the transverse part of 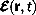, the boundary conditions are not restrictive enough: they allow for the variety of image charges (and therefore of surface-scattering models) discussed above.

Figures 1ii and 1iii show *ρ*_*a*_(**r**, *t*) and *ρ*_*b*_(**r**, *t*), respectively, for a case where the charge is in medium *a*. The Fourier transforms [Eqns (A1) and (A3)] of the charge distributions read


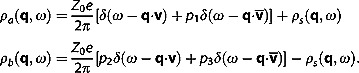
(9)

We now express the boundary conditions in terms of *ρ*_*a*_(**q**, *ω*) and *ρ*_*b*_(**q**, *ω*). First, the electric field and the displacement field will be expressed in terms of *ρ*_*a*_(**q**, *ω*) and *ρ*_*b*_(**q**, *ω*). Poisson's equation reads^25^,^26^


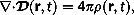
(10)

where *ρ*(**r**, *t*) is the distribution of external charges. In Fourier space, this equation becomes


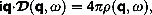
(11)

where *ρ*(**r**, *t*) is the distribution of free charge. Using Eqn (4) and the Fourier transform of Eqn (2),


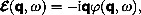
(12)

we can recast Eqn (11) as


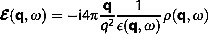
(13)

and write


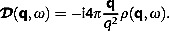
(14)

Taking the Fourier transform of the boundary conditions (5) and using Eqns (13) and (14), we have that the boundary conditions in terms of *ρ*_*a*_(**q**, *ω*) and *ρ*_*b*_(**q**, *ω*) are given by


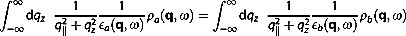
(15)

and


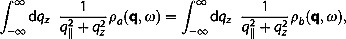
(16)

respectively. To derive the last two expressions, we have used the fact that the boundary conditions must hold everywhere on the surface at all times (∀ *x*, *y*, *t*). Notice that the dielectric functions at each side of the interface depend on **q** and *ω*. For the equalities to hold for all **q**_‖_ and *ω*, the charge densities must have a continuous dependency on **q** and *ω*. A collection of point charge distributions of the form *ρ*(**q**,*ω*) ∼ *δ*(*ω* − **q** ⋅ **v**) is therefore not enough to satisfy these two equalities for dispersive dielectric functions. An extended charge distribution with dependency on **q**_‖_ and *ω* (a surface charge distribution) is required, as we anticipated above.

The boundary conditions are now imposed. We start by inserting *ρ*_*a*_(**q**, *ω*) and *ρ*_*b*_(**q**, *ω*), Eqn (9), into Eqn (15). The fictitious surface charge distribution which renders the two infinite pseudomedia consistent with the semi-infinite geometry can be immediately isolated:


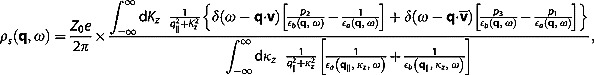
(17)

where in the numerator, **q** should be understood as (**q**_‖_, *K*_*z*_). The integral over *K*_*z*_ can be readily carried out using


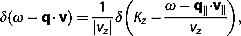
(18)


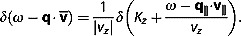
(19)

Defining


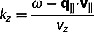
(20)

and


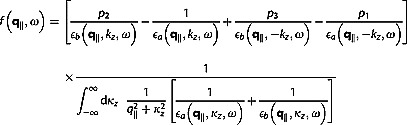
(21)

we can recast *ρ*_*s*_(**q**,*ω*) as


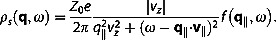
(22)

This expression coincides with Eqn (3) in Ref.^16^ within notation changes. The second boundary condition, Eqn (16), yields a closure relationship between *p*_1_, *p*_2_, and *p*_3_ which restricts the value of the fictitious point image charges:


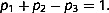
(23)

All literature models listed in Eqn (8) satisfy this relationship.

Once *ρ*_*s*_(**q**, *ω*) has been obtained, the total electric field, Eqn (13), is completely determined as


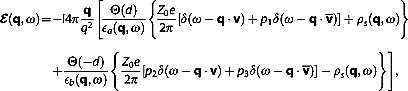
(24)

which can be recast more conveniently as


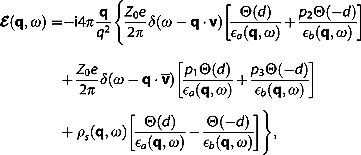
(25)

where Θ(*d*) is the Heaviside step function, which is 1 if *d* > 0 and 0 if *d* ≤ 0. The induced electric field, 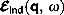, is obtained by subtracting the field that the projectile would create in vacuum, 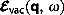, from the total electric field. From Eqn (13), we have that


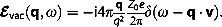
(26)

Thus, the induced electric field is given by


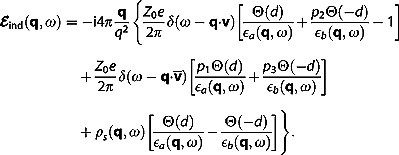
(27)

Finally, using Eqn (12), we have that the induced potential is given by


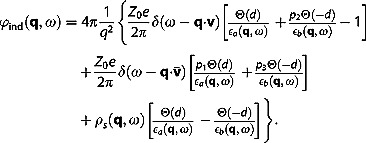
(28)

The induced electric field can be interpreted as the superposition of a field created by induced charges in the bulk of the media [first two terms in Eqn (27)] and a field created by an induced surface charge [third term in Eqn (27)]. Thus, the fictitious surface charge *ρ*_*s*_(**r**, *t*), which has so far served as a trick to satisfy the boundary conditions, is found to coincide with the induced surface charge.

### Induced surface charge

Before carrying on with the calculation of the stopping power, it is instructive to examine in some detail the induced surface charge *ρ*_*s*_(**r**, *t*) imposed by the boundary conditions, since all surface-related quantities will be obtained as integral transformations of *ρ*_*s*_(**r**, *t*) [see, e.g. Eqns (25) to (28)]. Thus, knowledge of the dependency of *ρ*_*s*_(**r**, *t*) on *d*, *η*, and **v** gives valuable insight into the conditions for which surface effects are to be expected.

The inverse Fourier transform of Eqn (22) reads


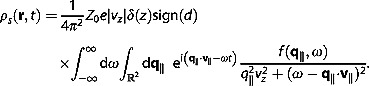
(29)

where we have carried out the trivial integration over *q*_*z*_ and where the term sign(*d*) has been explicitly introduced here to automatically select the appropriate sign of *ρ*_*s*_(**q**, *ω*) depending on whether the projectile is above or below the surface, see Fig. 1 and Eqn (7). With the change of variables 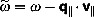, we have


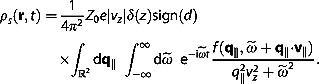
(30)

**Figure 2:**
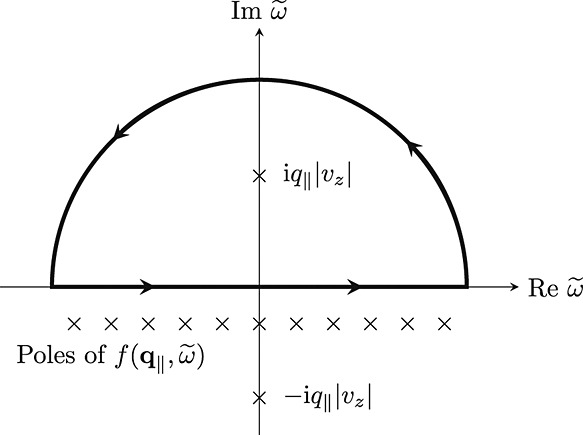
Integration contour used to carry out the integral over 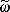 in Eqn (30).

Causality arguments imply that 1/*ε*(**q**,*ω*) is analytic in the upper half-plane of complex *ω*.^25^ Thus, *f*(**q**_‖_,*ω*) has all poles in the lower half-plane. For *t* < 0, we can therefore perform the integration over 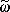 by closing the integration contour as indicated in Fig. 2 and using Eqns (B3) and (B8) to obtain


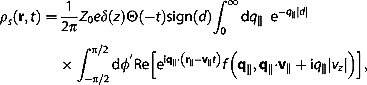
(31)

where **q**_‖_ = *q*_‖_(cos*ϕ*′, sin*ϕ*′, 0).

In the case *t* > 0, we consider Eqn (30) and use the second line of lemma (B1) and the property *ε*(**q**,*ω*) = *ε*^∗^(−**q**, − *ω*) to obtain


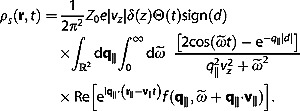
(32)

**Figure 3:**
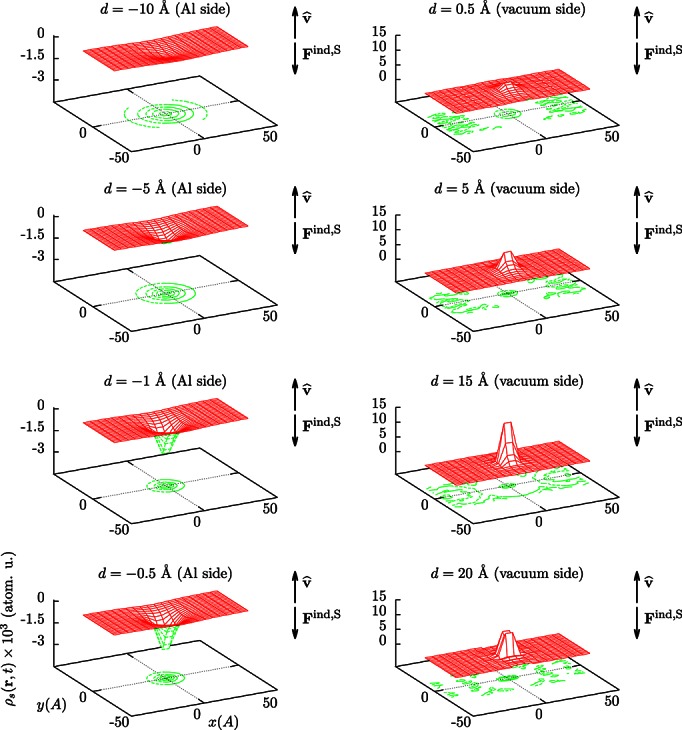
Surface polarization charge, Eqns (31) and (32), induced by a 500 eV electron that moves from Al into vacuum perpendicularly to the surface (*η* = 0) at different stages of its trajectory.

Figure 3 displays the surface polarization charge *ρ*_*s*_(**r**, *t*), Eqns (31) and (32), induced by an outgoing 500 eV electron that moves from Al (*d* < 0) to vacuum (*d* > 0) perpendicular to the interface (*η* = 0) for different values of the distance to the interface, *d*. The dielectric-function model used to produce this figure is given below, Eqn (66). The figure should be read by columns in order to follow the time evolution of *ρ*_*s*_(**r**, *t*) at different stages of the trajectory of the electron. The black arrows with the label 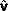 represent the direction of motion of the electron. When the electron approaches the interface from the Al side (*d* < 0), the induced surface charge *ρ*_*s*_(**r**, *t*) is negative, and therefore a repulsive force is induced on the electron. This force, schematically represented in the figure by the arrows labeled **F**_ind,S_, opposes the direction of motion of the electron: the electron is decelerated. Similarly, when the electron is at the vacuum side (*d* > 0), *ρ*_*s*_(**r**, *t*) is positive, and therefore an attractive force is induced on the electron. This attractive force opposes the direction of motion of the electron: the electron is also decelerated at the vacuum side. Thus, the contribution of the induced surface charge to the total induced force on an outgoing projectile moving perpendicularly to the surface results in a deceleration at both sides of the interface.

**Figure 4:**
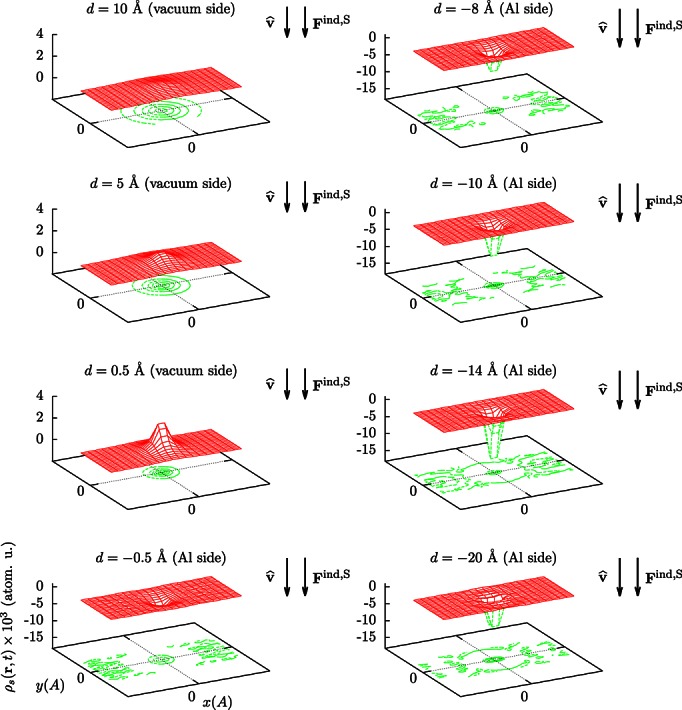
Same as Fig. 3 for *η* = 180^∘^ (incoming trajectory perpendicular to the surface).

In Fig. 4, we consider the opposite trajectory: an incoming electron with an energy of 500 eV crosses the interface from the vacuum side (*d* > 0) to the Al side (*d* < 0) in a direction perpendicular to the surface (*η* = 180^∘^). As the electron approaches the surface from the vacuum side (*d* > 0), a positive charge is induced at the surface, which creates an attractive force on the electron. This force is now along the direction of motion: the electron is accelerated. When the electron has crossed the interface into the Al side (*d* < 0), a negative charge is induced on the surface. Thus, a repulsive force **F**_ind,S_ acts on the electron. This force is also along the direction of motion: the electron is accelerated. Therefore, the contribution of the induced surface charge to the force induced on an incoming projectile moving perpendicularly to the surface results in an acceleration at both sides of the interface.

Regarding the effect of the induced surface charge on the probing projectile in the vacuum side, we can conclude the following: when a charged projectile approaches the interface to the medium perpendicularly, it is accelerated towards the surface. Conversely, when the projectile crosses the interface from the medium into the vacuum side, it is decelerated as it leaves the surface behind. This description is in accordance with Ref.^35^ Regarding the medium side, it should be noted that there is an additional contribution to the induced force from the induced bulk charges. We anticipate that, as we will see below, the acceleration and deceleration of the projectile cannot be directly translated into energy gains or losses.

**Figure 5:**
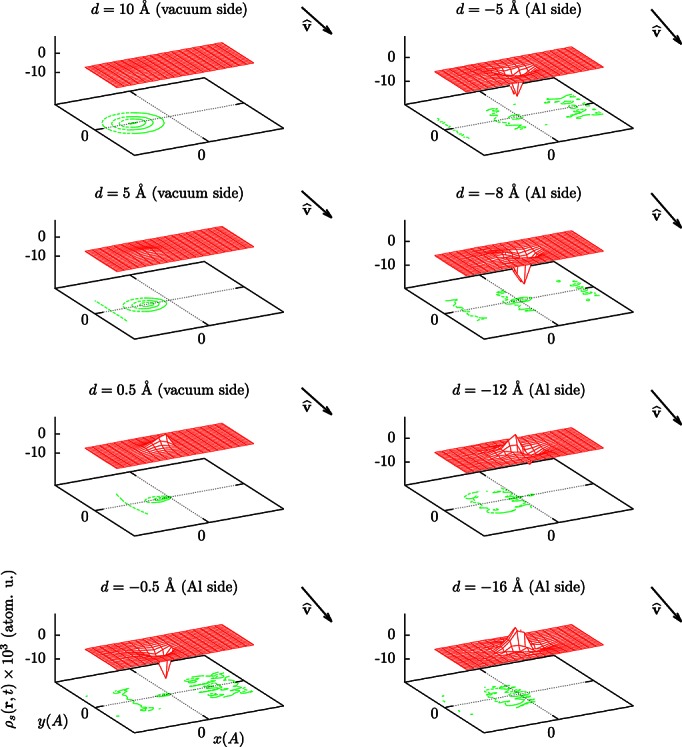
Same as Fig. 3 for *η* = 120^∘^ (oblique incoming trajectory).

In Fig. 5, we repeat the calculation for an oblique incoming trajectory with *η* = 120^∘^. A general behavior is observed in *ρ*_*s*_(**r**, *t*) in the three studied cases (*η* = 0, *η* = 120^∘^, and *η* = 180^∘^). As the projectile approaches the interface (regardless from which side), the magnitude of *ρ*_*s*_(**r**, *t*) increases monotonically. The projectile carries on with its motion, crosses the surface and, as it leaves the interface behind, the magnitude of *ρ*_*s*_(**r**, *t*) does not decrease monotonically: it exhibits an oscillating behavior which is eventually damped out as the projectile moves further away from the interface. Consequently, the force induced on the projectile is also expected to oscillate as the projectile moves away from the surface. Such oscillatory behaviors will be seen repeatedly in the following sections. In the studied cases, the magnitude of *ρ*_*s*_(**r**, *t*) is substantially different from zero only within a very thin layer of a width in the order of 1 nm or 2 nm around the interface. Therefore, surface effects are to be expected only in a very thin layer around the interface of, at most, a few nm. This also illustrates the surface sensitivity of contemporary surface-analysis techniques: surface characteristics originating from a layer of a few Angstroems within the surface are resolvable. We point out how remarkable it is that the method of image charges allows one to use the bulk dielectric functions of the media to describe processes that happen in a truly limited region of space.

### Stopping power

In order to gain a better insight into the dynamics of a charged projectile in the vicinity of an interface, in this section, we examine in detail the variation of its kinetic energy *E* per unit time:


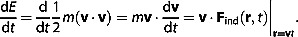
(33)

Following Ref.,^29^ this equation can be trivially recast as


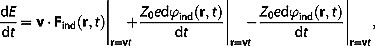
(34)

which merely states that the variation in kinetic energy is the variation in the total energy (kinetic plus potential, first two terms) minus the variation in potential energy (third term). On the other hand, the variation in kinetic energy of the projectile is given by the work *W* done on the projectile per unit time, which, in general, is the sum of a conservative and a dissipative contribution:


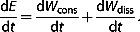
(35)

The conservative contribution d*W*_cons_/d*t* is given by the work done by the induced field, which is indeed conservative because it is the gradient of a potential:


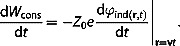
(36)

This conservative work can be recovered in principle by returning the projectile to its original configuration. The remaining two terms in Eqn (34) constitute the dissipative part of the variation in kinetic energy per unit time:


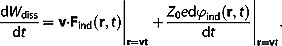
(37)

This dissipated work is by definition not recoverable at later stages of the trajectory; it is invested in an irreversible way in energy losses (or gains in very exceptional circumstances, see below) of the projectile in the excitation of certain modes of the solid. Thus, it is precisely this part of the kinetic energy variation that is needed to sample genuine energy losses of the projectile in a Monte Carlo simulation. Consequently, in what follows, we will concentrate on the analysis of the dissipative part of the variation of the kinetic energy. Carrying out the full time derivative in Eqn (37), we obtain


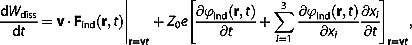
(38)

where {*x*_1_, *x*_2_, *x*_3_} are {*x*, *y*, *z*}, respectively. Thus, we have


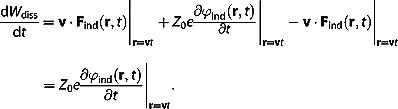
(39)

The variation due to the acceleration or deceleration caused by **F**_ind_(**r**, *t*) (first term) is exactly cancelled out by the third term. Therefore, the conclusions drawn in the previous section regarding the acceleration or deceleration of the projectile should not be translated directly into an increase or a decrease in the kinetic energy, i.e. into non-recoverable energy losses or gains. It is the implicit time dependency of *φ*_ind_(**r**, *t*) which ultimately determines the dissipated kinetic energy.

The approach we have followed (disregarding the conservative part of the variation in kinetic energy) is strictly valid for swift projectiles only. For these projectiles, the contribution of the conservative part along a reflected trajectory or a trajectory passing through a slab vanishes, provided that the velocity is not substantially modified during the interaction with the solid.

Incidentally, we notice that in an infinite medium, there is space and time invariance and, thus, the induced potential evaluated at the position of the projectile cannot exhibit a dependency on time. Therefore, the dissipative part of the variation in the kinetic energy, Eqn (37), reduces to **v** ⋅ **F**_ind_(**r**, *t*). Moreover, the conservative part of the variation in the kinetic energy, Eqn (36) vanishes. Under these special circumstances one can conclude whether the projectile undergoes an irreversible energy loss (or gain) alone from the direction of **F**_ind_(**r**, *t*). In the case of a semi-infinite geometry, Eqn (39) must be used.

The stopping power *S* is defined as the energy loss per unit path length,


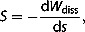
(40)

where d*s* = *v*d*t*. This quantity has dimensions of force. Using Eqn (39), we have


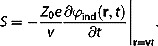
(41)

In terms of the Fourier transform of the induced potential, *φ*_ind_(**q**, *ω*), we have


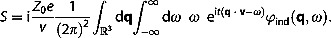
(42)

Using Eqn (28), we have that the stopping power is given by


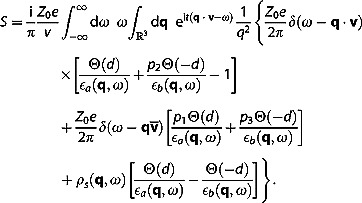
(43)

The first, the second, and the third summand are the stopping power of the projectile due to the interaction with the induced charges in the bulk, with the image charge (if selected), and with the induced surface charge, respectively. We will refer to these contributions using the symbols *S*_*B*_, *S*_*P*_, and *S*_*S*_, respectively. In what follows, we shall concentrate on the surface model with (*p*_1_, *p*_2_, *p*_3_) = (0,1,0), which implies setting the second term of Eqn (43) to zero. This approach then encompasses the models of Refs.^16^,^17^,^21^,^23^ The effect of the image charge, and to what extent it reproduces other models in the literature, will be discussed in a future work. See also the note after Eqn (8).

Let us first determine the stopping power due to the interaction with the induced bulk charges:


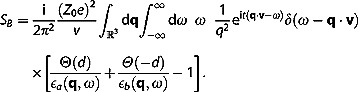
(44)

Assuming that *ε*(**q**, *ω*) = *ε*(*q*, *ω*), which is typically the case in practice, the integral over the azimuthal angle of **q** can be trivially carried out to yield 2*π*. The delta function can be used to carry out the integral over the cosine of the polar angle, *ξ*. A factor 1/*qv* appears from the change of variables in the argument of the delta function and, since − 1 ≤ *ξ* ≤ 1, the values of *ω* range from − *qv* to *qv*. Finally, using the property *ε*(**q**, *ω*) = *ε*^∗^(−**q**, − *ω*), we obtain


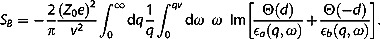
(45)

In an infinite medium, we would have only one of the dielectric-function terms, and we would retrieve the well-known expression for the stopping power in a bulk medium, see e.g. Eqn (1) of Ref. 15

Similarly, for the stopping power due to the interaction with the induced surface charge, we have


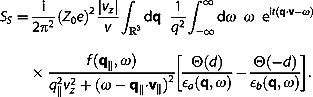
(46)

Using the property *ε*(**q**, *ω*) = *ε*^∗^(−**q**, − *ω*), we can recast this expression as


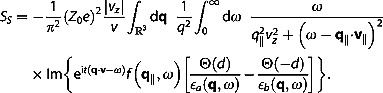
(47)

For times *t* < 0, we can carry out the contour integration over *ω* as described in the previous section. We define 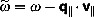 and recast Eqn (46) as


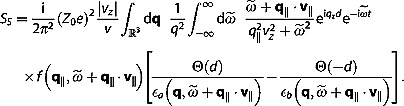
(48)

We close the integration contour through the upper half-plane in 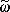 and obtain


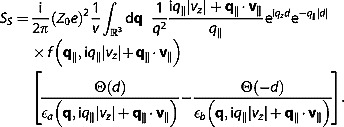
(49)

Using property 3, we obtain the final expression for the stopping power due to the interaction of the charged projectile with the induced surface charge:


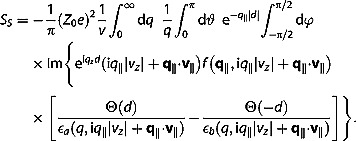
(50)

for *t* < 0. For *t* > 0, we use Eqn (47).

**Figure 6:**
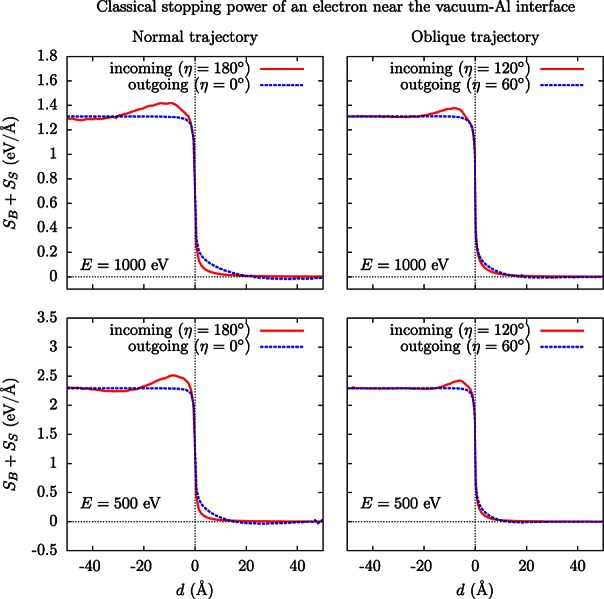
Sum of the classical stopping power due to the interaction with the induced bulk and surface charges (no image charges) for an electron near the vacuum–Al interface as a function of the distance to the interface, Eqns (45), (47), and (50). Here, *d* < 0 implies the Al side of the interface, whereas d > 0 implies the vacuum side.

Figure 6 displays the sum of the stopping power due to the interaction with the induced bulk and surface charges, Eqns (45), (47), and (50), for an electron traveling near the vacuum–Al interface as a function of the distance to the interface. We consider a normal trajectory (left-hand panels) and an oblique trajectory (right-hand panels). In both cases, we consider the incoming and the outgoing trajectories. The stopping power for the incoming (outgoing) trajectory is displayed by the solid (dashed) lines. As we noted when discussing the induced surface charge, oscillatory behaviors appear when the electron has left the interface behind (both for incoming and for outgoing trajectories). Thus, as is well-known, there is an asymmetry in the stopping power between the incoming and the outgoing trajectories: these differences become more important for trajectories crossing the surface with a direction close to the surface normal.

The negative oscillation found in the stopping power for the outgoing trajectory in the vacuum side of the interface would strictly imply that the projectile gains energy in a certain range of distances at the vacuum side of the sample. However, the stopping power integrated over all positive distances‡ is positive and, thus, we can assume that the projectile always loses energy, thereby introducing only minor errors. In the medium side of the interface, the stopping power is always positive, and, therefore, the projectile is always slowed down in the medium.

The magnitude of the stopping power at the vacuum side of the surface is generally smaller for the incoming trajectory than for the outgoing trajectory. A plausible explanation for this fact in connection with the conclusions of the previous section is the following. We recall that at the vacuum side of the incoming trajectory, the electron is accelerated towards the sample. Thus, the incoming electron spends less time in the vacuum side, and, therefore, less energy is lost in the incoming trajectory than in the outgoing trajectory.

### Semiclassical approximation

In the previous section, the stopping of the charged projectile has been attributed to the interaction with the induced electric field, and therefore considered as a continuous energy-loss process. In reality, however, the stopping of the charged projectile occurs in the course of consecutive discrete inelastic interactions with the loosely bound electrons of the medium. The link between the classical and the quantized descriptions is made by means of the so-called *semiclassical approximation*, which consists in identifying the Fourier variables **q** and *ω* with a discrete momentum transfer *ħ***q** and an energy loss *ħω*, respectively, in the course of an inelastic interaction. It is now convenient to use the system of atomic units; *ħ* = *e* = *m*_*e*_ = 1, where *m*_*e*_ is the mass of the electron. Since in atomic units, the energy loss *ħω* reduces to *ω*, we use the same symbol *ω* for the Fourier frequency and for the energy loss.

The integration domains of **q** and *ω* are now determined by the kinematics of inelastic collisions. Let the kinetic energy of the projectile be *E* before the collision and *E* − *ω* after the collision. Let **p** and 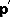, respectively, denote the wavevector of the charged projectile before and after the collision. The momentum transfer **q** is defined as


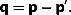
(51)

Taking the square at each side of this expression, we obtain


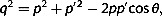
(52)

where *θ* is the polar scattering angle. In the non-relativistic case, energy and momentum are related by *E* = **p**^2^/(2*m*), namely 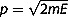, where *m* is the mass of the projectile. Thus, we can recast Eqn (52) as


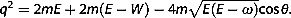
(53)

If the projectile is an electron, we have (*m* = 1):


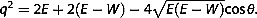
(54)

The maximum and minimum momentum transfer, *q*_+_ and *q*_−_, given by


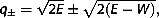
(55)

are achieved for *θ* = *π* and *θ* = 0, respectively. Thus, the integration domain of *q* will be restricted to [*q*_−_,*q*_+_]. This domain is valid for an electron both in an infinite and in a finite geometry: to derive it, we have merely used the definition of **q** and of the polar scattering angle. However, this does not imply that the azimuthal scattering angle is in general homogeneously distributed. Finally, the range of integration of the energy losses *ω* must be restricted to [0, *E*].

Thus, in the semiclassical approximation, the stopping power due to the interaction with the charges induced in the bulk is given by


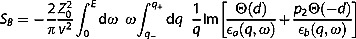
(56)

and the stopping power due to the interaction with the induced surface charge is given by


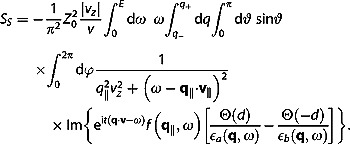
(57)

The Fourier phase might oscillate wildly and is therefore susceptible of posing numerical stability problems. In order to get rid of this problem, at least partially, we use lemma (B1) to recast *S*_*S*_ as


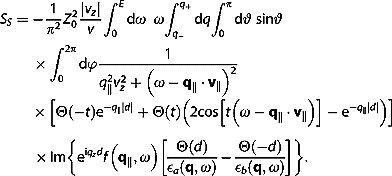
(58)

### DIIMFP

In a quantum-mechanical calculation, one would derive a differential interaction cross section for the inelastic scattering of the charged projectile with the electrons of the medium, d*σ*_*i*_/d*ω*d**q**. The quantity (d*σ*_*i*_/d*ω*d**q**)d*ω*d**q** is proportional to the probability that the charged projectile undergoes an inelastic interaction with an energy loss between *ω* and *ω* + d*ω* and a momentum transfer within d**q** of **q**. The double differential inverse mean free path (DDIIMFP) is defined as


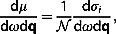
(59)

where 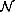 is the average density of electrons in the medium. The quantity (d*μ*/d*ω*d**q**)d*ω*d**q** is proportional to the probability that the charged projectile undergoes an inelastic interaction with an energy loss between *ω* and *ω* + d*ω* and a momentum transfer within d**q** of **q** per unit path length.

The electronic stopping power *S* is defined as the average energy loss per unit path length. To calculate it from the DDIIMFP, we would proceed as follows:


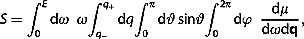
(60)

where the angles *θ* and *φ* are the polar and azimuthal angles of the momentum transfer in the Cartesian system of reference given in Fig. 1. Comparison of Eqn (60) with Eqns (56) and (58) allows us to identify


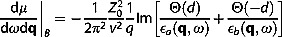
(61)

and


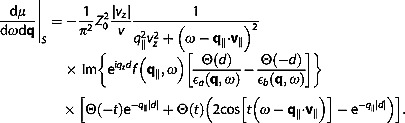
(62)

These are the contributions to the DDIIMFP due to the interaction of the charged projectile with the induced charges in the bulk and at the surface, respectively.

According to Eqn (53), the value of *q* is completely determined once a value for the energy loss *ω* and the polar scattering angle *θ* is fixed. Hence, for a fixed primary energy, the DDIIMFP is a function of the energy loss and of the polar and the azimuthal scattering angles (the kinetic energy *E*, the depth *d*, and polar angle *η* are parameters). The Cartesian components of the momentum transfer as a function of *E*, *ω*, *η*, and of the polar and azimuthal scattering angles *θ* and *ϕ* are given by:^37^


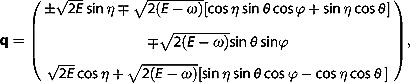
(63)

where the upper (lower) signs must be taken if *η* < 90^∘^ (*η* > 90^∘^).

**Figure 7:**
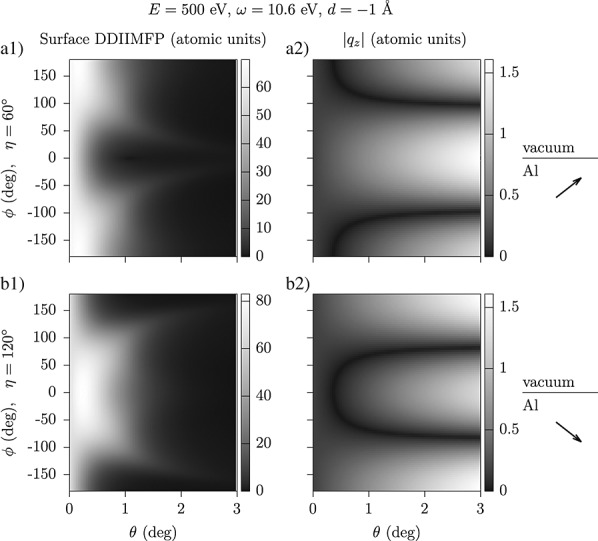
(a1 and b1) Contribution to the DDIIMFP due to the interaction with the induced surface charge [Eqn (62) using third component in Eqn (63)] for a 500 eV electron at a distance *d* = − 1 Å below the vacuum–Al interface for an energy loss *ω* = 10.6 eV as a function of the polar and azimuthal scattering angles, *θ* and *φ*, for off-normal polar angles *η* = 60^∘^ (a1) and *η* = 120^∘^ (b1). (a2 and b2) Absolute value of the momentum transfer perpendicular to the surface as a function of *θ* and *φ* for the scenarios corresponding to panels a1 and b1, respectively.

Figure 7 displays the contribution to the DDIIMFP due to the interaction with the induced surface charge [Eqns (62) using third component in Eqn (63)] as a function of the polar and the azimuthal scattering angles *θ* and *ϕ* for the following scenario: a 500 eV electron at a distance *d* = − 1 Å at the Al side of the vacuum–Al interface moving with an off-normal angle *η* = 60^∘^ (panel a1) and *η* = 120^∘^ (panel b1). We consider a fixed energy loss *ω* = 10.6 eV, corresponding to the excitation of a surface plasmon in Al. In this scenario, the DDIIMFP is significantly different from zero only for polar scattering angles which are less than ∼ 2^∘^. For higher energies, the DDIIMFP peaks at even smaller polar scattering angles. Hence, deflections in individual inelastic interactions can be neglected to a first approximation for *E* = 500 eV. Furthermore, notice that the azimuthal scattering angle is not distributed uniformly, as opposed to the case of an unlimited medium.

In panels a2 and b2, we plot the absolute value of the momentum transfer in a direction perpendicular to the surface, |*q*_*z*_|, as a function of the polar and azimuthal scattering angles. There is a strong anticorrelation between the magnitude of |*q*_*z*_| and the intensity of the DDIIMFP: momentum transfers along the surface (scattering angles such that |*q*_*z*_| ∼ 0) are strongly enhanced. Similar behaviors are found for other distances *d* at both sides of the interface, for other off-normal angles *η*, and for other materials.

Integration of the DDIIMFP over the kinematically allowed momentum transfers **q** gives a quantity that is proportional to the total distribution of energy losses in an inelastic interaction per unit path length. We will refer to this quantity as the DIIMFP and will use the symbol d*μ*/d*ω* to refer to it. The area under the DIIMFP curve gives a quantity with units of inverse length, known as the inverse inelastic mean free path (IIMFP), 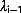. Its inverse, the inelastic mean free path (IMFP), *λ*_i_, gives the average distance to the next inelastic interaction.

From Eqn (61), we have that the contribution to the DIIMFP due to the interaction of the charged projectile with the induced bulk charges is given by


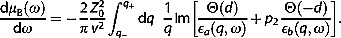
(64)

Similarly, from Eqn (62) we have that the contribution to the DIIMFP due to the interaction of the charged projectile with the induced surface charges is given by


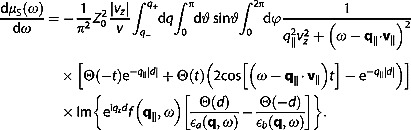
(65)

Thus, we have expressed the DIIMFP of a charged projectile moving close to a planar interface in terms of the dielectric functions of the media at each side of the surface, of the velocity **v** of the projectile, of the distance to the interface, *d*, and of the polar angle of the surface-crossing direction, *η*.

All expressions derived in the present work require the bulk dielectric function of the medium as input information. The user can naturally select whichever dielectric-function model is more appropriate for his problem. In the results below, we have used a classical-oscillator-model dielectric function, using two different parametrizations. In the case of Al, we have used


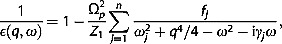
(66)

where *Z*_1_ is the atomic number of the target material, *f*_*j*_ are the oscillator amplitudes (dimensionless), 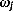 are the oscillator frequencies (in eV), and *γ*_*j*_ are the damping coefficients (in eV). The quantity 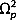 is the squared plasma resonance frequency of the material. In the case of Al, we have used a single-oscillator model with a resonance at *ω*_1_ = 15.01 eV, amplitude *f*_1_ = 3, and damping coefficient *γ*_1_ = 0.5 eV. For the 17 elemental metals listed in Ref.,^38^ we have used the dielectric-function model described therein with the supplied parameter values. It should be noted that the fit parameter values given in this reference are those which give the best agreement between the computed DIIMFP and the DIIMFP retrieved from a deconvolution procedure from pairs of REELS spectra: a good agreement should be obtained between simulated and experimental REELS spectra, at least regarding the bulk losses. Finally, notice that other dielectric-function models can prove more realistic in practice, such as the Mermin dielectric function.^39^ The reader is referred to Ref.^40^ for a discussion of the effect of different dielectric-function models on the calculation of bulk energy-loss functions.

## Results

**Figure 8:**
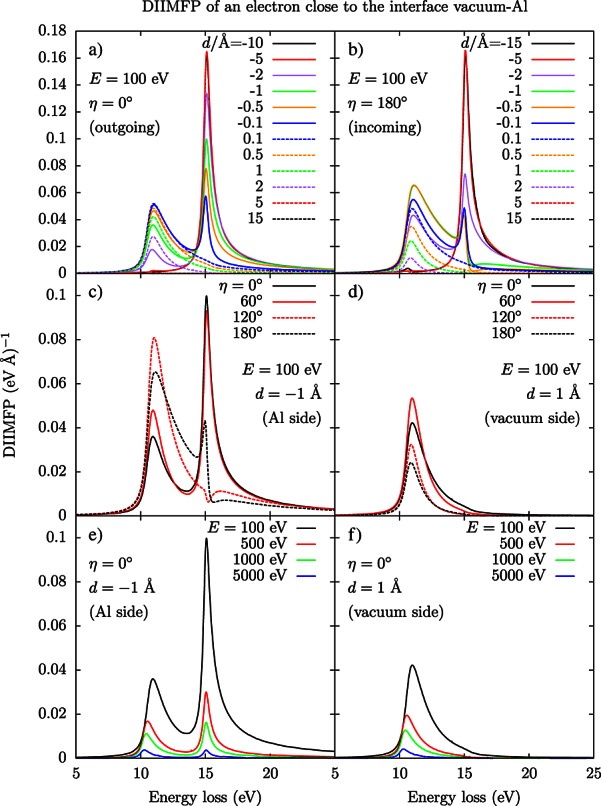
DIIMFP for an electron near the Al–vacuum interface, sum of Eqns (64) and (65). Examples for different kinetic energies *E*, depths *d*, and polar angles *η*.

Figure 8a displays the variation of the DIIMFP with *d* for a 500 eV electron moving perpendicularly from Al to vacuum (outgoing trajectory with *η* = 0^∘^), Eqns (64) and (65). When the electron is deep inside the Al side of the interface (solid black curve), the DIIMFP exhibits a peak at 15 eV, the excitation energy of a bulk plasmon in Al. As the electron approaches the interface (solid red to solid blue curves), the bulk-plasmon peak is progressively suppressed while a peak is enhanced at 10.6 eV, the excitation energy of a surface plasmon in Al. Thus, the effect of a planar interface is twofold: (i) it makes additional energy-loss modes available and (ii) it moves intensity from the bulk to the surface energy-loss features. The latter effect is known in the literature as the Begrenzung effect (German for delimitation). In a future work, the separation of the surface DIIMFP into a Begrenzung term and a clean surface term for the presented model will be studied in more detail. When the electron crosses the interface into the vacuum side (dashed curves in Fig. 8a), the bulk-plasmon peak is strongly suppressed, whereas the surface-loss peak prevails. This peak is in turn progressively suppressed as the electron moves further away from the interface (dashed blue through dashed black curves). The effect of the surface on the DIIMFP is noticeable in a region of about 10 Å at either side of the interface. In Fig. 8b, the contents of Fig. 8a for the incoming trajectory (*η* = 180^∘^) are reproduced. We see again that energy losses are possible at the vacuum side and that as the projectile moves deeper into the medium, surface-loss features are progressively suppressed to finally obtain bulk loss features.

Let us consider the DIIMFP for the case *d* = 2 Å (dashed purple curve), both in Fig. 8a (outgoing trajectory, interface already left behind) and 8b (incoming trajectory, interface not crossed yet). The DIIMFP is larger in the case of the outgoing trajectory, for which the electron has already left the interface behind. This is also the case for other values of *d* at either side of the interface and conclude that the probability of undergoing a surface loss (which will be precisely defined below) is enhanced once the projectile has left the interface behind (at either side).

Figure 8c and 8d illustrates the dependency on the polar angle of the trajectory, *η*. We consider a 100 eV electron at *d* = − 1 Å (in Al) and *d* = 1 Å (in vacuum), respectively. The dependency on *η* is not negligible: it is responsible for differences of up to 100%, and it modifies the relative height of the bulk and surface peaks. The surface-loss peaks are indeed enhanced for those values of *η* corresponding to trajectories which have already crossed the interface: *η* > 90^∘^ for *d* = − 1 Å and *η* < 90^∘^ for *d* = 1 Å.

**Figure 9:**
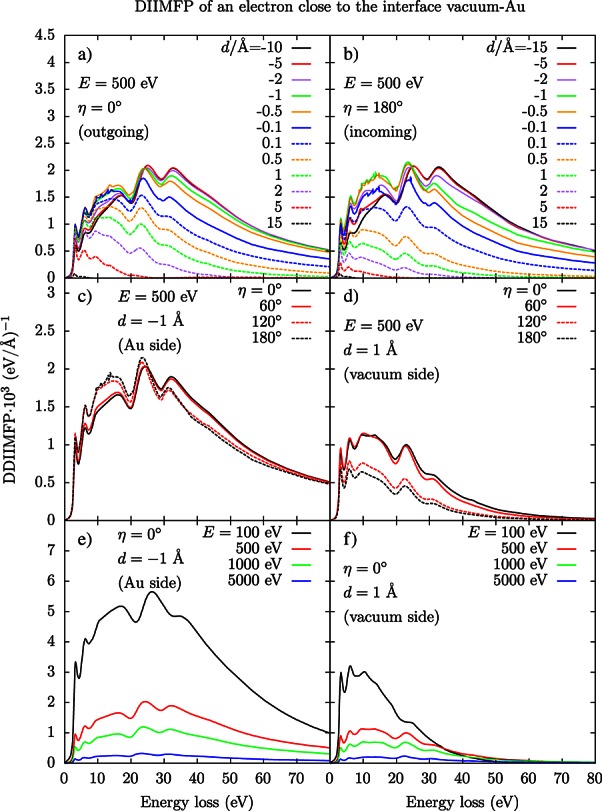
Same as Fig. 8 for Au and *E* = 500 eV in the two upper rows.

Finally, Figs. 8e and 8f exhibits the dependency of the DIIMFP on *E* for *η* = 0^∘^ and *d* = − 1 Å (Al side) and *d* = 1 Å (vacuum side), respectively. In the Al side, the relative height of surface and bulk peaks varies significantly with *E*. In the vacuum side, we clearly see that the surface losses are enhanced for low kinetic energies. We can attribute this to the naive observation that for low energies, the projectile spends more time crossing the surface-scattering region. Figure 9 displays a similar overview plot for the DIIMFP of an electron moving close to the vacuum–Au interface. The same conclusions apply.

### Comparison with literature models

In Ref.,^17^ a similar calculation was carried out to describe energy losses of charged projectiles near a planar interface (we point out that the approach was rather different, in that a trajectory-dependent effective cross section was derived). The following simplification was made:

‘To obtain the last expression, it has been assumed that […] ε(**q**,ω) = ε(**q**_‖_,ω) […]. Although the validity of this approximation is not clear, it is needed to obtain an analytical expression.’

This approximation (substitution of q by q_‖_ in the denominator of the expression for the induced surface charge density, Eqn (21)) will hereafter be called the Yubero *et al.* approximation. It was originally introduced for practical reasons in order to obtain tractable expressions which could allow for an easy calculation of electron energy-loss properties near surfaces. To the best of our knowledge, no detailed discussion of the implications or the effect of this approximation exists in the literature. Moreover, several later models^21^,^23^,^41^ use this approximation with the sole justification of a reference to the aforementioned work or to works which in turn make a reference to it. Another approximation that is made in some works (sometimes tacitly) consists in replacing **q** by **q**_‖_ in the last bracket in Eqn (65). We shall refer to this approximation as the **q** → **q**_‖_ approximation.

**Figure 10:**
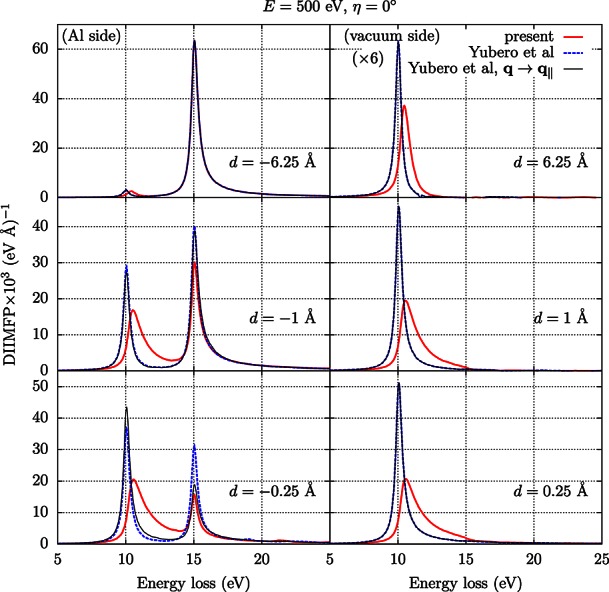
DIIMFP of an outgoing 500 eV electron moving perpendicularly through the Al–vacuum interface evaluated at different depths *d*. The red curves have been calculated with the model described in this work, Eqns (64) and (65). The blue curves employ the Yubero *et al.* approximation. The red curves, additionally, use the **q → q_∥_** approximation. The values of *d* are selected so that the thin black curve (Yubero *et al.* and **q → q_∥_** approximations) can be compared with Fig. 2 of Ref.^16^

Figure 10 shows the DIIMFP of a 500 eV electron moving perpendicularly from Al (*d* < 0) to vacuum (*d* > 0) for different distances *d* to the interface. The solid red curves are calculated using Eqn (65), whereas the dashed blue lines were calculated using the Yubero *et al.* approximation in the evaluation of the denominator of *f*(**q**_‖_, *ω*), and the thin solid black curves were calculated using both the Yubero *et al.* approximation and the **q** → **q**_‖_ approximation. The values of *d* were chosen so that the thin black curves can be compared with Fig. 2 of Ref.^16^

The effect of the Yubero *et al.* approximation is threefold: (i) it shifts the surface-loss peak to slightly smaller energy losses, ∼ 0.6 eV in the considered case; (ii) it enhances the surface and the bulk losses by as much as 50% and 75%, respectively; (iii) it reduces the variance and the skewness of the surface-loss peak. The effect of the **q** → **q**_‖_ approximation is significant only for very close distances to the interface at the medium side. It enhances the surface losses (up to ∼ 20 %) and suppresses the bulk losses (up to ∼ 50 %). Finally, in the vacuum side, the **q** → **q**_‖_ approximation has no effect, since *ε*(**q**, *ω*) = 1 for any **q**. Notice that, in essence, both the Yubero *et al.* approximation and the **q** → **q**_‖_ approximation imply replacing **q** by **q**_‖_. We recall that, as we saw in the previous section, the momentum transfer in an inelastic collision takes place predominantly in directions along the surface. Thus, we conclude that both the Yubero *et al.* and the **q** → **q**_‖_ approximations amount to an artificial enhancement of the surface-loss features. Finally, the difference between the DIIMFPs calculated with Eqn (65) and those calculated using the aforementioned approximations can be of the order of 100% in the worst cases, e.g. the lower panels of Fig. 10. Notice that the differences in the position, shape, and width of the peaks in the DIIMFP will ultimately affect the quality of the agreement between simulated and experimental REELS spectra. The number of surface or bulk excitations, however, will depend only on the area under the peaks, which does vary (but not drastically) when using each of the examined approximations.

In what follows, it will be shown how to derive the results of, e.g. Ref.^16^ from the general expressions given above. The contribution to the stopping power due to the interaction with the induced surface charge, *S*_*S*_, will be compared with Eqns (10) and (11) in Ref.^16^ We consider Eqn (58) without restricting the integral to the kinematically allowed region:


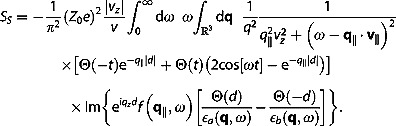
(67)

Introducing the notation changes given in Appendix C, Eqn (67) becomes Eqn (C3), which can be recast more conveniently as


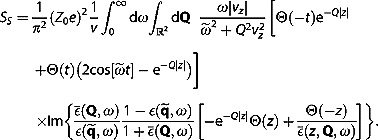
(68)

At this point, in order to reproduce Eqns (10) and (11) of Ref.^16^ we must assume that sign(*t*) = sign(*z*). This only holds for projectiles moving towards more positive values of *z*. Thus, we have


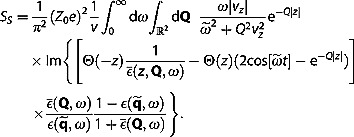
(67)

This expression matches Eqns (10) and (11) of Ref.^16^

Thus, the model in Ref.^16^ has the following restrictions:

- As pointed out by its authors, it is valid for outgoing projectiles, since we have had to impose sign(*t*) = sign(*z*). In recent works^21^,^23^ a distinction is made between trajectories which move from the solid to the vacuum (labeled *s* → *v*) and from the vacuum to the solid (labeled *v* → *s*).
- The integral over *q*_*z*_ is carried out from − *∞* to + *∞*. This disregards the fact that there is a minimum and a maximum momentum transfer which are kinematically allowed in an inelastic interaction. As a result, arbitrarily large momentum transfers are included in the integral. Conservation of energy and momentum is therefore not completely satisfied.
- In Fig. 10, in order to reproduce the figures in Ref.,^16^ both the Yubero *et al.* approximation and the **q** → **q**_‖_ approximation have had to be employed.

Although this model is not the current state of the art, it is still occasionally used in recent works,^42^ and it is simple enough so that it can be derived and examined within the present calculation in a straightforward way. More detailed models exist, such as the model presented by Li *et al.* in Ref.,^23^ which makes use of both the Yubero *et al.* and the **q** → **q**_‖_ approximation and, moreover, it allows for a convenient separation of the surface contribution to the DIIMFP in a begrenzung term (suppression of bulk excitation modes) and a true surface-excitation term. A comparison between the model of Ref.^23^ and the model presented here is not straightforward and has been omitted for the sake of brevity: this comparison will be addressed in the forthcoming second part of this work. The performance of Li's model has been assessed by Novák,^43^,^44^ obtaining a remarkably good agreement between simulated and experimental REELS spectra.

It should be emphasized again that the presented model comprises several models of the literature. By choosing appropriate values for (*p*_1_, *p*_2_, *p*_3_), e.g. (0,1,0) for the Chen and Kwei model, and introducing further approximations as required, it is in principle possible to reproduce most of the subset of semiclassical models for surface excitations found in the literature.^21^,^23^,^41^ As pointed above, the model of Yubero and Tougaard^17^ is not suited for comparison within the presented framework, for it takes a different approach. We do, however, point out that the Yubero and Tougaard model does account for the so-called interference effect between the outgoing electron and the field it set up upon entering the solid,^22^ whereas the model presented here does not. However, as shown by Vicanek,^22^ the interference effect might be significant for individual trajectories, but for a statistical ensemble of trajectories, for which the path-length distribution is usually much broader than the characteristic length for which interference effects play a role, the impact of these effects on the cumulative spectra is negligible. Last but by no means least, the agreement between the presented model and the Tung model^15^ shall be investigated in the upcoming second part of this work. This model, albeit simple in that it is not position dependent, is nevertheless of great use for practical applications in spectrum analysis.

We point out that another class of models for the energy loss of charged projectiles near planar surfaces have been developed within a quantum many-body calculation such as those of Ding.^19^,^20^ Their agreement with experimental data has been assessed,^45^ and their outcomes have been compared with those from models derived within the semiclassical dielectric formalism^46^ such as the Li model^23^ exhibiting a very good agreement except at surface-crossing angles larger than 89^∘^, implying that for most practical purposes, the semiclassical and the quantum computation are equivalent. These models have not been considered in the present discussion, where we have concerned ourselves with derivations within the semiclassical dielectric formalism.

## Monte Carlo algorithm for the simulation of REELS spectra

In this section, we describe a Monte Carlo algorithm for the simulation of the transport of charged projectiles in a semi-infinite geometry. See, e.g. Ref.^37^,^47^ for the sampling algorithm in the absence of surface excitations. In the present case, the procedure is very similar to the method described by Ding and Shimizu^45^ and is based on the sampling algorithm of Coleman.^48^ It is assumed that a database of DIIMFPs for different materials, primary energies *E*, polar angles *η*, and depths *d* has been calculated on a grid of points of *E*, *η*, and *d* dense enough so that for a given material, a trilinear interpolation on *E*, *η*, and *d* is sufficient to interpolate values of the IMFP and the DIIMFP. Moreover, the differential cross section for elastic scattering (DCES), d*σ*_e_/d*Ω*, is assumed to be calculated for a grid of energies *E* which covers the range of interest for the simulation and which is dense enough so that linear (lin-lin or lin-log) interpolation suffices. The IMFP *λ*_i_(*E*,*d*,*η*) and the elastic mean free paths *λ*_e_(*E*) are tabulated beforehand.

The core of the algorithm reads

1. Find the minimum mean free path among all processes (elastic scattering for all *E*, inelastic scattering for all *E*, *d*, *η*), *λ*_min_.
2. Initialize the trajectory (position and direction). In the case of (R)EELS, the primary electron is initialized at the highest position above the sample for which there are database entries. For XPS or AES, the electron is initialized at the corresponding emission depth. Notice that in the case of XPS, intrinsic losses due to the interaction of the photoelectron with the core-hole left behind are not accounted for by the present model.
3. If the electron is in the surface-scattering region (between the minimum and the maximum *d* for which there are database entries):

- Take a step 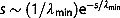 along the current direction.
- Interpolate *λ*_i_ and *λ*_e_ at the current kinetic energy, position, and direction of flight (*λ*_e_ = 0 at the vacuum side).
- Sample an event of the type:
  - null with probability 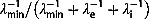.
  - elastic interaction with probability 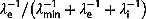.
    - Let *E*_*i*_ ≤ *E* < *E*_*i* + 1_. Sample a deflection from 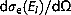 or 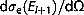 with probabilities
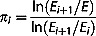
(70)
      and
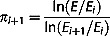
(71)
      respectively.^47^
    - Update the direction of flight of the electron.
  - inelastic interaction with probability 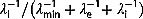. We now need to sample an energy loss from the database for the current *E*, *η*, and *d*. Let *E*_*i*_ ≤ *E* < *E*_*i* + 1_, *η*_*j*_ ≤ *η* < *η*_*j* + 1_ and *d*_*k*_ ≤ *d* < *d*_*k* + 1_. Let *X* denote *E*, *η*, or *d* and let the subscript *α* denote the corresponding subscript *i*, *j*, or *k*, as needed. We take *X* = *X*_*α*_ or *X* = *X*_*α* + 1_ with probabilities

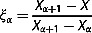
(72)

and


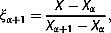
(73)

respectively. Let *α*_*i*_, *α*_*j*_, and *α*_*k*_ denote the chosen value for the subscripts *i*, *j*, and *k*, respectively. Once the active subscripts *α*_*i*_, *α*_*j*_, and *α*_*k*_ are determined, we sample an energy loss from 
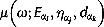
 and update the energy of the projectile.

Else, if the projectile is outside of the surface-scattering region:

- Interpolate *λ*_i_ and *λ*_e_ at the current energy. Calculate the total mean free path 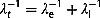.
- Take a step 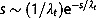 along the current direction.
- Sample an event of the type:

- elastic interaction with probability 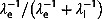.
  - Let *E*_*i*_ ≤ *E* < *E*_*i* + 1_. Sample a deflection from 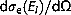 or 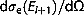 with probabilities *π*_*i*_ and *π*_*i* + 1_ defined above, respectively.
  - Update the direction of flight of the electron.
- inelastic interaction with probability 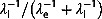.
  - Let *E*_*i*_ ≤ *E* < *E*_*i* + 1_. Sample an energy loss from *μ*(*E*_*i*_) or *μ*(*E*_*i* + 1_) with probabilities *π*_*i*_ and *π*_*i* + 1_ defined above, respectively.
  - Update the energy of the electron.

4. If the electron leaves the sample (surface-scattering region) without entering the analyzer or if the electron leaves the energy window of interest, disregard the trajectory and sample the next one. Otherwise, sample the current trajectory further by going back to step 3. If the electron leaves the sample in a direction within the solid angle of detection around the detector direction, update the corresponding histograms.

### Comparison with experimental REELS spectra

The algorithm outlined in the previous section has been used to simulate a database of experimental REELS spectra^49^–^51^ for 24 elemental solids measured on a VG Microlab UHV system equipped with a hemispherical analyzer, used with a constant resolution mode with a pass energy of 20 eV. The beam of incident electrons impinged normally on the sample and the analyzer formed a polar angle of 60^∘^ with the surface normal. The pressure during the measurements was in the 10^− 9^ mbar range. See Refs^49^–^51^ for more details. The experimental spectra were converted to absolute units of 1/eV through a division by the area under the elastic peak and by the bin width.

The database of DIIMFPs and DCESs required for the simulation has been generated as follows. The optical data provided in Ref.^38^ has been used to generate the required database of *E*-, *η* -, and *d*-dependent DIIMFPs, using Eqns (64) and (65). We have used the following grid: *E* = {100,500,1000,5000} eV, *η* = {0^∘^,60^∘^,120^∘^,180^∘^}, *d* = {±15, ± 5, ± 2, ± 1, ± 0.5, ± 0.1} Å. Additionally, a database of 50 bulk DIIMFPs has been generated for energies equally spaced on a logarithmic grid from *E* = 100 eV to *E* = 10 keV. DCESs for 150 energies equally spaced on a logarithmic scale from *E* = 100 eV to *E* = 10 keV have been generated with the ELSEPA code,^52^ using a muffin-tin model potential obtained from the Dirac-Fock electron density, with exchange effects accounted for by means of the Furness-McCarthy exchange potential. Correlation-polarization and inelastic-absorption corrections have not been considered.

**Figure 11:**
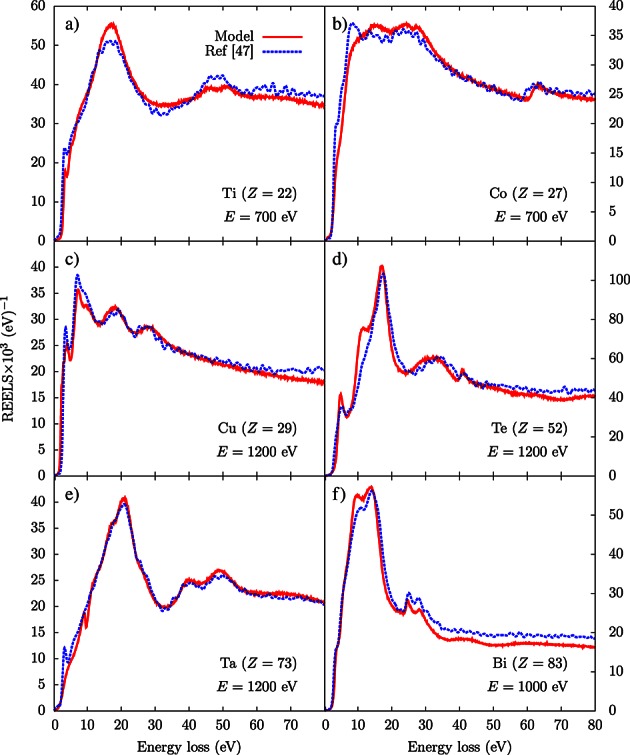
Simulated REELS (solid curve) compared with experimental REELS (dashed curve). Note that the comparison is in absolute units (homogeneous renormalization by the number of elastically detected electrons). Optical data taken from Ref.^38^

The simulation of the aforementioned database of experimental REELS spectra (as well as the calculation of the DIIMFP database) was carried out at the Vienna Scientific Cluster, using Intel Xeon X5550 (Nehalem) processors with a 2.97 GHz clockspeed. The sampling algorithm attained an average speed of 5 × 10^4^ up to 10^5^ simulated trajectories per second; 10^9^ trajectories were sampled for each REELS spectrum with a resolution of 0.1 eV. In Fig. 11, we compare six of the simulated REELS spectra with their experimental counterparts, with primary energies from 700 eV to 1200 eV and metals ranging from Ti (*Z* = 22) to Bi (*Z* = 83). The solid curves are the simulated spectra, whereas the dashed curves are the measured spectra. Notice that the comparison is in absolute units of reciprocal eV: the experimental spectra have been divided by the area of the elastic peak (which was subsequently subtracted) and by the bin width; the simulated spectra have been divided by the number of elastically detected electrons and by the bin width. The simulated spectra are in good agreement with the experimental spectra, the discrepancies being between 5% and 10% in the considered energy-loss range. The agreement is strongly dependent on the quality of the used optical data, to a much higher degree than in the simulation of bulk losses only. Finally, we point out that the intensity scale of the normalized experimental spectra might be off by ∼ 5% due to the uncertainty in the determination of the area under the elastic peak. We point out that, although the primary energies in Fig. 11 vary between 700 eV and 1200 eV, a similarly good agreement was found for other energies.

As outlined above, the algorithm requires considerable computational effort. If only bulk losses are considered, the trajectory reversal algorithm^53^ can be employed to drastically decrease the simulation time. However, this algorithm relies on the symmetry of the loss characteristics between the incoming and the outgoing trajectory. This symmetry is lost in the surface-excitation model described here. The trajectory-reversal algorithm can therefore not be employed to accelerate the simulation. In a future work, it will be discussed to what extent the present model can be used to derive a cumulative surface-loss distribution (the so-called differential surface excitation probability (DSEP)), an approach similar to that of Ref.^15^ DSEP-type loss distributions can be easily embedded in the partial-intensity approach^54^ for a simplified and yet accurate description of surface excitations in electron spectroscopy.

**Figure 12:**
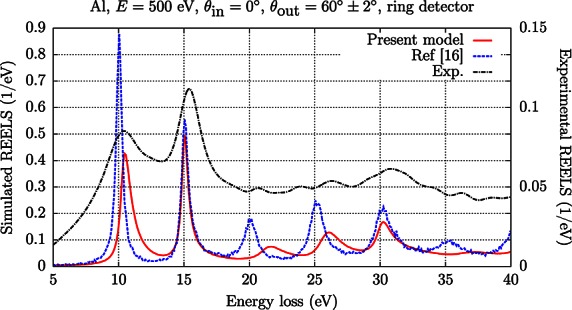
Simulated REELS spectrum of 500 eV electrons impinging perpendicularly on a polycrystalline Al sample detected by a ring detector at 60^∘^ ± 2^∘^ using the model described in this work (solid curve) and the model in Ref.^16^ (dashed curve, see text). The dot-dashed curve corresponds to an experimental REELS spectrum measured for the same geometry (with a HMA at 60^∘^ off-normal angle with a half-opening angle of ∼ 12^∘^) and primary energy.

Finally, in Fig. 12, the effect of the Yubero *et al.* and the **q** → **q**_‖_ approximation on simulated REELS spectra is exposed. We consider the REELS spectrum of 500 eV electrons impinging perpendicularly on an Al surface with a ring detector accepting outgoing trajectories with off-normal polar angles between 58^∘^ and 62^∘^. The solid curve was calculated using the model described in this work, whereas the dashed curve was calculated using the model presented in Ref.^16^ (the model presented here was used with the approximations addressed above to match the model in Ref.^16^ The database of calculated DIIMFPs comprised the following parameter values: *E* = {450,500}, *η* = {0,60,120,180}, *d* = {±15, ± 5, ± 2, ± 1, ± 0.5, ± 0.1}. The differences we observed in the DIIMFPs between the two models are naturally translated into the REELS spectrum: surface features are accentuated and shifted towards smaller energy losses, and the relative intensity of bulk and surface losses is modified. An experimental REELS spectrum of 500 eV impinging normally on Al and being detected along a polar angle of 60 ± 12^∘^ is shown. The relative bulk/surface height and the asymmetry and width of the surface-loss peaks are well described by the present model. Regarding the intensity scale and the overall sharpness of the simulation results, it must be pointed out that the simulation of REELS for Al is typically problematic with the employed dielectric-function model: the DIIMFPs derived from optical data usually give too narrow loss features in comparison with the experimental loss features. Similar difficulties are found for other nearly free-electron metals such as Si. Nevertheless, they are ideal candidates for a benchmark of model calculations due to their sharp loss features, both in the bulk and in the surface. A convolution with a normalized Gaussian peak of the estimated experimental energy resolution might lead to a change in the relative intensity of surface and bulk peaks. We note that the employed dielectric-function model does not properly include electron-hole pair generation. The use of other dielectric-function models which account for electron-hole pair generation such as the Mermin dielectric function^39^,^40^ should lead to a better agreement between simulated and experimental REELS spectra for nearly free-electron materials such as Al and Si.

## Conclusions

A comprehensive and self-contained description of the dynamics of non-relativistic charged projectiles in the vicinity of a planar interface is provided within the dielectric formalism, considering only the longitudinal part of the force induced on the projectile. The presented framework allows one to derive a differential IIMFP (DIIMFP) of a charged projectile as a function of its speed, of its direction of motion, and of its distance to the interface. The derivation has been carried out in such a way that (i) it encompasses a number of models in the literature and (ii) unnecessary simplifications are avoided. The effect of the assumptions and simplifications made in literature models has been investigated, revealing differences of up to 100% (in the worst cases) in the intensity of the DIIMFP, as well as appreciable differences in its shape. DIIMFPs calculated with the model described here have been used as input for a Monte Carlo algorithm for the simulation of REELS spectra, obtaining good agreement between simulated and experimental spectra in absolute units. We therefore conclude that the presented model can be applied in a detailed simulation of energy-loss processes of charged projectiles near planar interfaces.

## Footnotes

† Although in practice the medium above the surface will often be taken as vacuum, with *ε*_*a*_(**q**,*ω*) = 1, we keep an arbitrary medium *a* to retain generality.

‡ Note that this integration is only meaningful for very fast projectiles.

## Appendix A: Fourier transform

We shall use the following definition of the direct and inverse Fourier transform and its inverse, using symmetrical factors of  for each of the four integration variables:

(A1)

(A2)

As an example, consider the Fourier transform of a charge distribution *ρ*(**r**,*t*) = *Z*_0_*eδ*(**r** − **v***t*):

(A3)

where we have used a familiar representation of the delta distribution.

## Appendix B: Mathematical tools

Lemma 1

If *f*(*ω*) is a function of *ω* with no poles in the upper complex half-plane, then

(B1)

For *t* ≤ 0, the lemma follows from direct integration by residues of the integrands at both sides of the equality along a semi-circular path on the upper half-plane, picking up the pole at *ω* = i*a*. For *t* > 0, we can use the trivial identity

(B2)

and close the integration contour again along a semicircle on the upper half-plane.

1. Properties of the dielectric function for complex arguments

When carrying out contour integrals over *ω*, it becomes necessary to evaluate *ε*(**q**,*ω*) and *f*(**q**_‖_,*ω*) for complex values of *ω*. The properties of these functions for complex *ω* will now be examined. Since in all practical cases, we consider *ε*(**q**,*ω*) = *ε*(*q*,*ω*), we shall restrict ourselves to a classical-oscillator dielectric-function model of the form of Eqn (66).

Property 1

For a complex frequency of the form *ω* = i*q*_‖_|*v*_*z*_| + **q**_‖_**v**_‖_, we have

(B3)

*Proof:* For *ω* = **q**_‖_**v**_‖_ + i*q*_‖_|*v*_*z*_|, we have

(B4)

If we change **q**_‖_ by − **q**_‖_, we have

(B5)

□

Property 2

(B6)

*Proof:* From the definition of *f*(**q**_‖_,*ω*), we have that

(B7)

□

For *ω* = i*q*_‖_|*v*_*z*_| + **q**_‖_**v**_‖_, we have that *k*_*z*_ = i*q*_‖_sign(*v*_*z*_). Therefore, .

Property 3

For a complex frequency of the form *ω* = i*q*_‖_|*v*_*z*_| + **q**_‖_**v**_‖_, we have

(B8)

provided that *ε*(**q**,*ω*) = *ε*(*q*,*ω*).

□

*Proof:* It follows directly from Eqn (B3) and the definition of *f*(**q**_‖_,*ω*).

## Appendix C: Notation changes in the literature

We introduce the following notation changes in order to comply with Ref.: 16

(C1)

With this new notation, we have

(C2)

Thus,

(C3)
